# Impaired vision in children prenatally exposed to methadone: an observational cohort study

**DOI:** 10.1038/s41433-023-02644-3

**Published:** 2023-07-04

**Authors:** R. Hamilton, A. Mulvihill, L. Butler, A. Chow, E. Irving, D. L. McCulloch, A. McNeil, K. Michael, K. M. Spowart, J. Waterson-Wilson, H. Mactier

**Affiliations:** 1 Royal Hospital for Children, NHS Greater Glasgow & Clyde and the University of Glasgow, Glasgow, G51 4TF UK; 2 Princess Alexandra Eye Pavilion, NHS Lothian, Edinburgh, EH3 9HA UK; 3 Tennant Institute of Ophthalmology, NHS Greater Glasgow & Clyde, Glasgow, G12 0YN UK; 4 School of Optometry and Vision Science, University of Waterloo, Waterloo, Ontario N2L 3G1 Canada; 5 Royal Hospital for Children, NHS Greater Glasgow & Clyde, Glasgow, G51 4TF UK; 6 Crosshouse Hospital, NHS Ayrshire & Arran, Kilmarnock, KA2 0BE UK; 7 Specialist Children’s Services, NHS Greater Glasgow & Clyde, Glasgow, G40 1DA UK; 8 NHS Greater Glasgow & Clyde and the University of Glasgow, Glasgow, UK

**Keywords:** Paediatrics, Epidemiology

## Abstract

**Background/objectives:**

To examine prevalence of failed visual assessment at 8–10 years in children born to methadone-maintained opioid dependent (MMOD) mothers and relate this to known in utero substance exposure.

**Subjects/methods:**

Follow up of observational cohort study of methadone-exposed and comparison children matched for birthweight, gestation and postcode of residence at birth. Participants were 144 children (98 exposed, 46 comparison). Prenatal drug exposure was previously established via comprehensive maternal and neonatal toxicology. Children were invited to attend for visual assessment and casenotes were reviewed. Presence of acuity poorer than 0.2 logMAR, strabismus, nystagmus and/or impaired stereovision constituted a ‘fail’. Fail rates were compared between methadone-exposed and comparison children after adjusting for known confounding variables.

**Results:**

33 children attended in person: data were also derived from casenote review for all children. After controlling for maternal reported tobacco use, methadone-exposed children were more likely to have a visual ‘fail’ outcome, adjusted odds ratio 2.6, 95% CI 1.1–6.2; adjusted relative risk 1.8 (95% CI 1.1–3.4). Visual ‘fail’ outcome rates did not differ between methadone-exposed children who had (*n* = 47) or had not (*n* = 51) received pharmacological treatment for neonatal abstinence/opioid withdrawal syndrome (NAS/NOWS); fail rate 62% vs 53% (95% CI of difference—11–27%).

**Conclusions:**

Children born to MMOD mothers are almost twice as likely as unexposed peers to have significant visual abnormalities at primary school age. Prenatal methadone exposure should be considered in the differential diagnosis of nystagmus. Findings support visual assessment prior to school entry for children with any history of prenatal opioid exposure.

**Trial registration:**

The study was prospectively registered on ClinicalTrials.gov (NCT03603301), https://clinicaltrials.gov/ct2/show/NCT03603301.

## Introduction

Opioid use disorder (OUD) in pregnancy causes significant harm [1, 2]. Harm reduction policies, including maintenance treatment with methadone and/or other opioids, improve engagement with antenatal care and reduce risk-taking behaviours and preterm birth [1, 3, 4]. Prenatal opioid exposure may lead to neonatal abstinence/opioid withdrawal syndrome (NAS/NOWS); causation of longer-term sequelae for these children is difficult to ascertain due to confounding effects of often poorly documented polydrug misuse, tobacco smoking, alcohol and challenged home environment [5–9].

Prenatal opioid exposure has been associated with problems suggesting impaired development of vision [6, 10–36] including strabismus and nystagmus; reports approximately mirror global opioid epidemics [3, 37–39]. In a cohort of healthy infants born to methadone-maintained opioid dependent (MMOD) mothers, for whom extensive toxicology data were available, altered neonatal visual evoked potentials (VEP), an objective physiological marker of integrity and maturity of the visual pathway, were independently associated with prenatal methadone exposure [40]. Vision was impaired at six months of age, as well as general development [41, 42]. We now report follow-up data from this cohort at ages 8–10 years with the aims of comparing vision fail outcome rates between exposed and comparison children after controlling for confounding variables, and relating visual outcomes to prenatal substance exposure.

## Methods

### Design

This observational cohort study investigated visual outcomes of prenatally methadone-exposed children compared with comparison children, aged 8–10 years. The study was pre-registered at ClinicalTrials.gov, NCT 03603301.

### Participants

The cohort was previously recruited at birth and investigated at 1–3 days [40] and at 6–7 months of age [41]. Eligible exposed infants were born to MMOD mothers after 36 completed weeks’ gestation without congenital ocular abnormality or significant neonatal illness. Prenatal drug exposure was determined via comprehensive toxicology (maternal urine, infant urine and meconium), maternal casenote review and confidential interview [40]. Comparison infants were born contemporaneously (2008–10) at the same maternity hospital, matched for completed week of gestation, birthweight (±250 g) and material deprivation. Maternal postcode of residence at delivery was used to define the 2001 Carstairs material deprivation category (±1, 1–7 from least to most deprivation based on four indicators judged to represent material disadvantage (lack of car ownership, low occupational social class, overcrowded households and male unemployment) [43]). As far as possible, comparison infants were also matched for maternal tobacco use. A subgroup of comparison infants had meconium drug analysis and a subset of meconium samples from both groups was analysed for prenatal alcohol exposure. The exposed cohort size (*n* = 102, of whom 100 completed neonatal VEPs) was chosen for sufficient VEP parameter precision to distinguish exposed infants who developed NAS/NOWS (defined as receiving pharmaceutical treatment as per well-established hospital protocol) from those who did not [11]. Comparison cohort size (*n* = 51, of whom 50 completed neonatal VEPs) was chosen for pragmatic reasons given the time commitment required for neonatal testing and 6–7 month follow up. Selection bias was low due to the high consent rate (98%) at infant recruitment [40].

For follow-up at 8–10 years, families of all recruited children, including three who did not complete neonatal VEPs, were invited to attend for investigation. Initial contact was by letter asking families to opt in or out via a return slip; most recent home address was sought from general practitioners if there was no response. Follow-up phone calls were made to families who opted in and to non-responders.

### Measures

Children attending in person were assessed at the paediatric Clinical Research Facility, Queen Elizabeth University Hospital, Glasgow. This healthcare complex includes the regional children’s hospital and the regional paediatric ophthalmology service. Written informed consent was given by the accompanying adult; children gave written informed assent. Care status (living with birth parent(s), kinship carer, foster carer or adoptive parent(s)) as well as diagnoses of autistic spectrum disorder (ASD), attention deficit hyperactivity disorder (ADHD), and/or foetal alcohol spectrum disorder (FASD) were noted and details of any previous attendance at ophthalmology clinics were scrutinised. Following detailed visual assessment (see supplementary material), children were ascribed a ‘pass’ or ‘fail’ result based on pre-determined fail criteria including one or more of: acuity poorer than 0.2 logMAR not attributable to refractive error; manifest but not latent strabismus; nystagmus; abnormal stereovision. Visual assessment pass/fail was the primary outcome measure. To quantify the extent of visual problems a novel, composite ‘visual detriment index’ (VDI) was created for the study as a secondary outcome measure. Severity of fail criteria were scored (nystagmus = 3, strabismus = 2, impaired binocular vision = 2, poor acuity = 2) and summed, giving possible scores 0–9. A higher score was attributed to nystagmus because of its relatively higher detriment to vision and to reflect how uncommon it is in the population at large.

To avoid bias due to any comprehension difficulties, vision tests were selected to be easily performed by primary school age children. Researchers were masked to exposure status to limit bias potential. Reporting bias (carers of children with eye problems more likely to attend) would likely affect both exposed and comparison families similarly.

Children failing visual assessment or causing concern not already being addressed were notified to relevant services after discussion with their carer. Families were offered cash to cover expenses and a £20 child’s gift voucher.

For children who did not attend for follow up (no contact details, family did not respond to invitation, actively declined to attend in person or failed to attend arranged assessment(s)), casenotes were reviewed for record of any hospital eye service findings. Casenote review is a robust process due to two factors; 1) mandated use of the Community Health Index number in NHS Scotland which enables linking of health data for research purposes and 2) comprehensive, electronic-only patient health records for both acute and community settings. The same pre-determined vision ‘fail’ criteria were used and both care status and diagnoses of ASD, ADHD, and/or FASD noted if available. Any non-attending child confirmed to be living within the health board region (NHS Greater Glasgow & Clyde) at the time of casenote review but not ever referred to hospital eye services was attributed a ‘pass’ result. The Scottish universal pre-school vision screening programme ensures a high identification rate of visual problems (89% coverage for 2008–10 births) and subsequent referral to regional services. The likelihood of a ‘pass’ result being incorrectly attributed to a non-attending child was therefore felt to be low. Casenote review was also undertaken for children who attended in person.

### Analyses

Characteristics and outcomes of attendees versus non-attendees were compared to assess any bias associated with in-person prospective data collection versus data collected via retrospective casenote review. Agreement on pass/fail outcome between casenote review and in-person assessment was compared for attending children.

Visual assessment pass/fail was presented as an unadjusted odds ratio (ratio of fail outcomes, exposed vs comparisons, to ratio of pass outcomes, exposed vs comparisons). Odds ratio is a poor approximation of the more intuitive relative risk (risk ratio) for outcomes which are not rare [44] and so unadjusted relative risk was also calculated. To identify potentially confounding variables, maternal, birth and neonatal characteristics were compared between exposed and comparison groups. Methadone exposure and potentially confounding variables were treated as independent dichotomous variables (non-exposed as the reference category) in a multivariable logistic regression analysis with pass/fail visual outcome as the dependent variable, and in a multivariable linear regression analysis with VDI as the dependent variable. For exposed children only, visual outcomes were compared by drug exposure group, by presence or absence of (treated) NAS/NOWS and by prescribed maternal methadone dose at birth. Multivariable linear regression was used to examine the relationship between each additional drug exposure and VDI. Sex and care status were treated as potential modifiers for sub-group analysis. Based on the series of three research investigations of this cohort, the positive and negative predictive value of the first investigation (abnormal neonatal VEPs [40]) and the second investigation (a failed or borderline visual assessment at 6–7 months [41]) were calculated.

Analyses were performed using Minitab v20.3 (Mintab LLC, PA, USA) and MedCalc® v20.014 (MedCalc Software Ltd, Ostend, Belgium). The study was approved by West of Scotland Research Ethics Committee 3 (17/WS/0093).

## Results

### Participants

Of the whole cohort of 153 children, eight were untraceable; one further child was excluded due to a diagnosis of retinal dystrophy. Of the remaining 144 children (98 exposed, 46 comparison), 67 (46%) did not respond to invitations, 26 (18%) declined to attend in person, 18 (13%) did not show and 33 (23%) attended. Data were therefore derived from casenote review alone for 111 children (77 exposed, 34 comparison) and from findings at attendance as well as casenote review for 33 (21 exposed, 12 comparison) children (supplementary material Fig. S1). 45 of the 111 non-attending children (27 exposed, 18 comparison) were still resident in the health board region but had not ever been referred to its hospital eye services and thereby were attributed a ‘pass’ result.

Attendees and non-attendees did not differ in terms of birth characteristics, prenatal drug exposure, demographics or visual outcomes at last documented follow-up. Age at assessment was older for attendees than age at most recent hospital eye service attendance or other healthcare encounter for non-attendees (shown for exposed and comparison groups, supplementary material Table S1). Social care differed markedly between exposed and comparison children; over half (49/93) of exposed children no longer lived with either birth parent. Fourteen MMOD but no comparison mothers had died (Table 1). Two exposed children were homeless.

**Table 1:** Comparison of demographic and birth characteristics, maternal factors, social and care arrangements and visual findings for exposed versus comparison children.

	Methadone exposed children, *n* = 98 unless otherwise indicated	Comparison children, *n* = 46 unless otherwise indicated	Difference (95% CI of difference)	Test and *p* value
Age^a^, yr^b^	7.8 (2.3)	7.9 (2.1)	−0.07 (−0.8–0.7)	*t* 0.17, df=96, *p* = 0.86
Sex, (*n*) male	(45) 45.9%	(21) 45.7%	0.3 (−17–17)%	*χ*^2^ 0.001, df=1, *p* = 0.98
Mode of delivery, (*n*)				*χ*^2^ 1.003, df=2, *p* = 0.61
Spontaneous vertex delivery	(70) 71%	(31) 67%	4 (−11–21)%	
Lower uterine segment caesarean delivery	(22) 22%	(10) 22%	0.5 (−15–14)%	
Instrumental vaginal delivery	(6) 6%	(5) 11%	−5 (−17–4)%	
Gestation, week^c^	39.3 (38.3–40.1)	39.9 (38.4–40.8)	−2 (−6–1) days	MW 6825, *p* = 0.23
5-min Apgar^c^	9 (9–10)	9 (9–10)	0 (0–0)	MW 7123, *p* = 0.94
Birthweight, g^b^	2898 (509)	3078 (533)	−181 (368–6)	*t* 1.92, df=84, *p* = 0.058
Small for gestational age (< 3rd centile), (*n*)	(17) 17%	(9) 20%	−2 (−17–10)%	*χ*^2^ 0.103, df=1, *p* = 0.75
Low birthweight (< 2500 g), (*n*)	(19) 19%	(7) 15%	4 (−10–16)%	*χ*^2^ 0.365, df=1, *p* = 0.55
Occipitofrontal head circumference at birth, cm^b^	33.5 (1.6)	34.3 (1.6)	−0.8 (−1.4–0.3)	*t* 2.91, df=89, *p* = 0.005
Microcephaly (< 2nd centile) at birth, (*n*)	(8) 8%	(4) 9%	−0.5 (−8–13)%	Fisher exact, *p* = 1
Formula fed at discharge, (*n*)	(86) 88%	(41) 89%	−1 (−12–11)%	*χ*^2^ 0.056, df=1, *p* = 0.81
Maternal tobacco use, (*n*)	(93) 95%	(25) 54%	41 (26–55)%	*χ*^2^ 34.55, df=1, *p* < 0·0001
Reported cigarettes per day^c;a^	10 (10–15), *n* = 93	10 (10–20), *n* = 25	0 (0–0)	MW 5536, *p* = 0.99
Prenatal alcohol exposure, (*n*) %	(25) 39%, *n* = 59	(5) 25%, *n* = 20^d^	17 (−7–36)%	*χ*^2^ 1.89, df=1, *p* = 0.26
Maternal body mass index^c^	24 (21–26)	23.5 (21–30.25)	−1 (−3–1)	MW 6891, *p* = 0.36
Maternal Carstairs deprivation category^c^	7 (5–7)	6 (4.75–7)	0 (0–1)	MW 7365, *p* = 0.27
Pharmacologically treated neonatal opioid withdrawal syndrome, (*n*) %	(47) 48%			
Birth mother deceased, (*n*) %	(14) 14%	(0) 0%	14 (1–22)%	Fisher exact, *p* = 0.01
Adopted; foster/kinship care, (*n*) %	(49) 53%, *n* = 93	(6) 15%, *n* = 41	38 (21–51)%	*χ*^2^ 16.902, df=1, *p* < 0.0001
Attended hospital eye services, (*n*) %	(66) 67%	(18) 39%	28 (11–44)%	*χ*^2^ 10.183, df=1, *p* = 0.0014
Visual outcome ‘fail’, (*n*) %	(56) 57%	(12) 26%	31 (14–45)%	*χ*^2^ 12.031, df=1, *p* = 0.0005
Strabismus, (*n*) %	(46) 47%	(6) 13%	34 (18–46)%	*χ*^2^ 15.481, df=1, *p* = 0.0001
Poor acuity, (*n*) %	(25) 26%	(7) 15%	10 (−5–22)%	*χ*^2^ 1.906, df=1, *p* = 0.17
Poor binocular vision, (*n*) %	(22) 22%	(5) 11%	12 (−3–23)%	*χ*^2^ 2.736, df=1, *p* = 0.098
Nystagmus, (*n*) %	(20) 20%	(0) 0%	20 (10–29)%	Fisher exact, *p* = 0.001
Visual detriment index, VDI^c^	2 (0–4) range 0–9	0 (0–2) range 0–6	1 (0–2)	MW 7941, *p* < 0.0005

*CI* confidence interval, *df* degrees of freedom, *MW* Mann Whitney test.

^a^Age at assessment (*n* = 33 children) or age at most recent hospital eye service attendance (*n* = 66 children) or age at most recent healthcare encounter with confirmed local address but no hospital eye service encounters (‘pass’ vision result attributed, *n* = 45).

^b^Mean (standard deviation).

^c^Median (inter-quartile range).

^d^Due to data loss, prenatal alcohol exposure is known only for the five positive comparison children: denominator is unknown but estimated to be n = 20 based on neonatal data proportions.

**Fig. 1:**
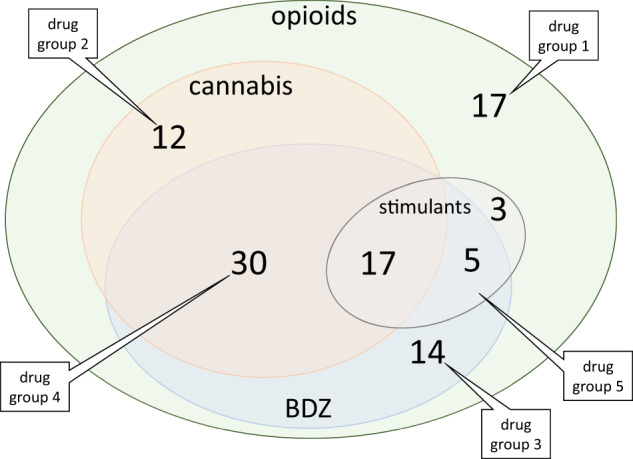
Euler diagram illustrating combinations of polydrug exposure and drug groups based on combined exposure data (see Table 1) for the 98 exposed children. Opioid is methadone ± opiates; BDZ, benzodiazepines; stimulants are cocaine and/or amphetamines. Drug groups: 1) opioids alone (*n* = 17); 2) opioids + cannabinoid (*n* = 12); 3) opioids + benzodiazepine (*n* = 14); 4) opioids + benzodiazepine + cannabis (*n* = 30); 5) opioids + stimulants ± benzodiazepine or cannabis (*n* = 25). Opiates most likely illicit heroin. If infant or postnatal maternal urine was positive for opiates and opioid analgesia in labour was documented, in the absence of declared illicit maternal opiate or positive prenatal maternal urine, infant was not considered exposed.

Maternal reported tobacco use differed significantly between exposed (93/98, 95%) and comparison children (25/46, 54%, Table 1) and was therefore treated as a potentially confounding variable. Head circumference at birth also differed significantly between exposed and comparison children (Table 1), but since prenatal opioid exposure is associated with reduced fetal head growth [6, 44], head circumference at birth was not treated as a confounding variable. Only 17/98 exposed children were prenatally exposed solely to opioids (drug group 1, Fig. 1, Table 2); for nine, this was exclusively prescribed methadone.

**Table 2:** Prenatal drug exposure (see Fig. 1).

	Exposed children *n* = 98					Comparison children *n* = 46			
Drug, (*n*) % positive	History *n* = 98	Maternal urine *n* = 80	Infant urine *n* = 66	Meconium *n* = 72	Combined *n* = 98	History *n* = 2	Infant urine *n* = 2	Meconium *n* = 26	Combined *n* = 28
Methadone	(97) 99%	(74) 93%	(40) 61%	(69) 96%	(98) 100%	(0)	(0)	(0)	(0)
Opiates^a^	(54) 55%	(46) 58%	(24) 36%	(55) 76%	(75) 77%	(0)	(2)^b^	(0)	(2)^b^
Benzodiazepine	(51) 52%	(48) 60%	(22) 33%	(38) 53%	(66) 67%	(0)	(1)	(0)	(1) 4%
Cannabis	(17) 17%	(30) 38%	(5) 8%	(46) 64%	(60) 61%	(1)	(1)	(1)	(3) 11%
Amphetamine	(2) 2%	(1) 1%	(0)	(10) 14%	(14) 14%	(1)	(0)	(1)	(2) 8%
Cocaine	(4) 4%	(4) 5%	(2) 3%	(10) 14%	(13) 13%	(1)	(0)	(1)	(2) 8%

^a^Opiates most likely illicit heroin.

^b^If infant or postnatal maternal urine was positive for opiates and opioid analgesia in labour was documented, in the absence of declared illicit maternal opiate or positive prenatal maternal urine, infant was not considered exposed.

### Visual findings

Visual pass/fail outcomes from in-person assessment and from casenote review matched for all 33 (100%) children attending for assessment. Children born to MMOD mothers were more likely than comparison children to have a visual ‘fail’ outcome: 56/98 vs 12/46 (Table 1). The unadjusted odds ratio, 3.8 (95% CI 1.8–8.2, *p* = 0.001), indicates children failing vision assessment were about four times more likely to have been born to MMOD mothers than children who passed. The unadjusted relative risk, 2.2 (95% CI 1.3–4.0), indicates that children born to MMOD mothers were over twice as likely to fail vision assessment. Exposed children were also more likely to have attended or be attending hospital eye services and had higher median VDI (Table 1). Twenty (20%, 95% CI 13–30%) exposed children had nystagmus, none with an explanatory diagnosis or family history. Sixteen of these 20 had previously been assessed at 6–7 months: nystagmus was evident in only eight (50%) at that age [41]. Six of these 20 children with nystagmus attended in person; eye movement recordings (supplementary material) showed waveforms consistent with fusion maldevelopment nystagmus syndrome. No comparison child had nystagmus.

After controlling for reported maternal tobacco use, children born to MMOD mothers were more likely to have a visual fail outcome (adjusted odds ratio 2.6, 95% CI 1.1–6.2, Table 3A). The adjusted relative risk of children born to MMOD mothers failing visual assessment at 8–10 years was 1.8 (95% CI 1.1–3.4). Similarly, being born to a MMOD mother was associated with significantly higher VDI after controlling for maternal tobacco use, with an adjusted effect size of 1.4 (95% CI 0.45–2.36) (Table 3B).

**Table 3:** Regression models.

**A**. Logistic multivariable regression model describing the association of failed visual outcome with methadone exposure before and after adjusting for maternal reported tobacco use									
	Unadjusted					Adjusted			
Independent variables	Coefficient (standard error)	Odds ratio (95% CI)	Relative risk (95% CI)		*p*	Coefficient (standard error)	Odds ratio (95% CI)	Relative risk (95% CI)	*p*
Methadone exposure	1.33 (0.39)	3.8 (1.7–8.2)	2.2 (1.3–4.0)		0.001	0.97 (0.43)	2.6 (1.1–6.2)	1.8 (1.1–3.4)	0.025
Maternal reported tobacco use	-	-	-		-	1.03 (0.58)	2.8 (0.9–8.8)		0.08
**B**. Linear multivariable regression model describing the association of visual detriment index (VDI) with methadone exposure before and after adjusting for maternal reported tobacco use									
	unadjusted					adjusted			
Independent variables	coefficient	95% CI	*p*			coefficient	95% CI	*p*	
Methadone exposure	1.73	0.89–2.56	<0.0005			1.40	0.45–2.36	0.004	
Maternal reported tobacco use	-	-	-			0.80	-0.36–2.0	0.17	
**C**. Multivariable linear regression parameters: association of drug exposures in addition to methadone with visual detriment index (VDI) in childhood (exposed children only, *n* = 98). *R*-squared 6.8%, *F* value 1.33, *p* = 0.26									
Drug exposure in addition to methadone				coefficient (standard error)			T	*p*	
Benzodiazepine				0.39 (0.64)			2.18	0.032	
Amphetamine				−1.23 (0.82)			−1.50	0.14	
Cocaine				−0.84 (0.81)			−1.04	0.30	
Cannabis				−0.10 (0.60)			−0.17	0.87	
Opiates				−0.01 (0.66)			−0.01	0.99	

*CI* confidence interval.

A) association of failed visual outcome and B) visual detriment index (VDI) with methadone exposure before and after adjusting for maternal reported tobacco use, and C) association of drug exposures in addition to methadone with visual detriment index (VDI) in exposed children.

**Fig. 2:**
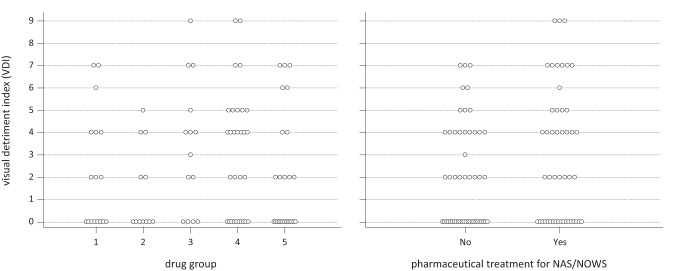
Dotplots showing visual detriment index (VDI) for 98 exposed children by drug group (left) and by treated neonatal abstinence/opioid withdrawal syndrome (NAS/NOWS) status (right). Left, by drug exposure group: 1) opioids alone (*n* = 17); 2) opioids + cannabinoid (*n* = 12); 3) opioids + benzodiazepine (*n* = 14); 4) opioids + benzodiazepine + cannabis (*n* = 30); 5) opioids + stimulants (cocaine and/or amphetamines) ± benzodiazepine or cannabis (*n* = 25). Right, by neonatal abstinence/opioid withdrawal syndrome (NAS/NOWS) treatment category (*n* = 47 treated, *n* = 51 not treated).

For children born to MMOD mothers, VDI did not differ by drug-exposure group (Kruskal-Wallis test, *H* = 6.3, df = 4, *p* = 0.18). The 17 opioid-only exposed children (drug group 1) with visual fail outcomes had similar findings to the exposed group as a whole (Fig. 2).

Visual fail outcome rates did not differ between exposed children who had (29/47, 62%) or had not (27/51, 53%) been treated for NAS/NOWS (95% CI of difference -11–27%, *χ*^2^ 0.8, df = 1, *p* = 0.4) and neither did VDI differ between children who had or had not been treated for NAS/NOWS (median 2 vs 2, Mann-Whitney W = 2527, *p* = 0.16, Fig. 2).

MMOD mothers of children failing visual assessment had been prescribed higher daily methadone doses at delivery than mothers of children who passed (57.5 vs 45 mg per day, 95% CI of difference 5–27 mg, Mann-Whitney 3008, *p* = 0.016). Prescribed maternal methadone dose at delivery was weakly and positively correlated with VDI (rho 0.31, 95% CI 0.11–0.49, *p* = 0.002).

Multivariable ordinal regression modelling of additional drug exposure(s) found only benzodiazepine to be independently associated with higher VDI (Table 3C), suggesting that prenatal benzodiazepine in addition to methadone exposure slightly but significantly further impairs visual outcomes.

Considering the whole cohort, a similar proportion of visual fail outcomes was found for male (35/66, 53%) and female (33/78, 42%) children (95% CI of difference −6–26%, *p* = 0.20). For children born to MMOD mothers, similar proportions of visual fail outcomes were found for children in kinship, foster or adoptive care (30/48, 63%) and for children living with a birth parent (24/44, 55%) (95% CI of difference −12–27%, *p* = 0.44): care status was therefore unlikely to be a significant modifier.

From the series of three research investigations of this cohort, 140 children had both neonatal VEP data and visual findings at 8–10 years: abnormal neonatal VEPs [40] had positive and negative predictive values for failed childhood visual outcome of 77 (61–88)% and 73 (60–84)% respectively. For the 102 children with both 6–7 month and 8–10 year visual findings, a failed or borderline result at 6–7 months [41] had lower positive and negative predictive values for failed mid-childhood visual outcome, 56 (42–69)% and 59 (48–70)%, respectively. Sixteen infants passed at 6–7 months but subsequently had a visual fail outcome childhood assessment, including three children with nystagmus.

## Discussion

Within this cohort, after adjusting for maternal tobacco use in pregnancy, being born to a MMOD mother almost doubled the risk of children failing visual assessment at 8–10 years. Almost half of exposed children had strabismus, conservatively 10-fold the expected prevalence of 2–3.4% in UK children [45, 46]. One in five exposed children had nystagmus, conservatively 300-fold the expected prevalence in children free of neurological or retinal disease [47]. Poorer outcomes were associated with higher prescribed maternal methadone dose and visual problems were equally frequent in children who did and did not receive treatment for NAS/NOWS. Research assessments of this cohort at 6–7 months [40, 41] were poorly predictive of childhood visual outcomes. However, all children with fail outcomes at 8–10 years had already been identified via routine healthcare provision.

More than half of children born to MMOD mothers with a visual fail outcome at 8–10 years had strabismus and/or nystagmus; this is more common than reported elsewhere, which may reflect higher ascertainment with universal access to pre-school vision screening. The high rate of strabismus even in our comparison children (13%) may be explained at least partly by maternal tobacco use. By comparison, 47% of exposed children had strabismus; the marked difference between the groups persisted even after controlling for maternal tobacco smoking. An alarmingly high proportion—20%—of exposed children had nystagmus with a waveform consistent with fusion maldevelopment nystagmus syndrome, suggesting impaired cortical visual input to subcortical vestibular pathways [48]. This may imply a teratogenic effect of methadone (and/or other substances of misuse) upon the striate cortex and connective tracts, reducing the number and/or connectivity of binocular connections [49, 50] which could explain both strabismus and fusion maldevelopment nystagmus syndrome [51]. Additional exposure to benzodiazepine was associated with more visual problems, suggesting that benzodiazepine slightly but significantly further impairs visual outcomes. This association did not hold for additional opiates, cannabis, cocaine or amphetamines.

A teratogenic effect of prenatal opioid exposure upon the developing fetal brain is both plausible and supported by evidence including smaller head size at birth and neonatal brain MRI showing loss of connective tracts [50], multiple observational studies [10–26], the albeit weak dose-response relationship with prescribed methadone dose at delivery in the current study, and evidence from animal models [52, 53]. Meta analyses also strongly suggest an association between prenatal methadone exposure and impaired childhood neurodevelopmental outcomes [5–7]. While all of these data support an association between prescription of methadone to opioid-dependent pregnant women and impaired childhood visual outcomes, they do not prove causation [36].

A cautious conclusion from this study is that children born to MMOD mothers are up to twice as likely as unexposed peers to have visual abnormalities by mid-primary school age. As previously noted in a much larger cohort, a history of NAS/NOWS is not required for the child to be at risk of longer term harm [32]. Current guidelines [1, 4] state that methadone is safe in pregnancy other than the risk of (transient) NAS/NOWS: while methadone maintenance therapy improves pregnancy outcomes, pregnant mothers and their caregivers need to balance management of OUD with consideration of the risk of longer term problems for the unborn child [32], which are not currently described in guidelines. Strabismus and especially nystagmus confer learning and psychosocial difficulties [54], additional burdens on children with reduced life opportunities [55].

From the visual findings described in this study and by others, as well as other neurodevelopmental problems [5, 6, 32], we propose a fetal opioid spectrum disorder and suggest that prenatal opioid exposure be considered in the differential diagnosis of infantile and/or childhood nystagmus. Even if children born to MMOD mothers remained well in the neonatal period, they should have formal visual assessment post-infancy and prior to school entry with emphasis on strabismus, binocular vision and nystagmus. Consideration of further detailed visual assessment is warranted, particularly if there are educational and/or behavioural difficulties. All health professionals caring for children have a role in ensuring these vulnerable children are assessed, especially when universal childhood visual screening programmes are lacking. Studies to determine the longer term safety of methadone alternatives, such as buprenorphine, should include visual follow up [24, 56–58] and should control for maternal tobacco use.

Strengths of this study include detailed knowledge of prenatal drug exposure, prospective design and a comparison group matched at recruitment for gestation, birthweight and deprivation. The additional challenges of drug misuse mean that postcode of residence is not a perfect proxy for socioeconomic deprivation, and tobacco smoking is also very difficult to control for. A major limitation of the study was the small proportion (23%) of the cohort attending for assessment. Significant efforts were made to contact families, the vast majority of whom remained resident in the Glasgow area. However, researching some of the most materially-deprived families in Scotland, many of whom have chaotic, unpredictable lifestyles and do not engage well with healthcare or perceived authority, is challenging and therefore in-person investigation of 33 of the traceable cohort of 144 children represents a substantial achievement. Furthermore, casenote review proved to be robust, matching in-person findings in terms of the primary outcome measure, visual assessment pass/fail. Tobacco use may be underestimated due to maternal self-report but with 95% reported use by MMOD mothers, this is likely to have applied only to comparison mothers of whom 54% reported tobacco use. Prenatal alcohol exposure was assessed by meconium analysis of only a subgroup of both exposed and comparison children, meaning less reliable assessment of this confounding factor. Assuming a ‘pass’ result for non-attending children confirmed to be living locally but not ever referred to hospital eye services may bias the findings, but bias is likely to be limited given the high retention of local residency and high coverage by the national pre-school universal vision screening programme. Since our cohort included exclusively a treatment population (children of MMOD mothers), our findings are not generalisable to children of other opioid users such as unsupported opiate users or those using licit opioids.

Being born to an MMOD mother is strongly associated with visual problems in mid-childhood. Pregnant women with OUD and their caregivers need to consider the potential risk of visual abnormalities and other developmental disorders in the unborn child. Prenatal opioid exposure should be considered in the differential diagnosis of nystagmus and, regardless of whether NAS/NOWS was manifest, children born to MMOD mothers should have formal visual assessment before school entry.

## Summary

### What was known before

- Treatment of opioid use disorder in pregnancy with methadone maintenance treatment improves pregnancy outcomes.
- Prenatal opioid exposure may lead to neonatal abstinence/opioid withdrawal syndrome (NAS/NOWS) and abnormal visual evoked potentials: longer-term sequelae are reported but research is confounded by polydrug misuse, tobacco smoking, alcohol and challenged home environment.
- Impaired visual development including strabismus and nystagmus has been reported but no long-term cohort data are available.

### What this study adds

- Being born to a methadone-maintained opioid dependent mother almost doubled the risk of children failing visual assessment at 8–10 years.
- Almost half of exposed children had strabismus and one in five had nystagmus. Visual problems were equally frequent in children who did and did not receive treatment for NAS/NOWS.
- Prenatal methadone exposure should be considered in the differential diagnosis of nystagmus.
- Findings support visual assessment prior to school entry for children with a history of prenatal exposure to opioids and/or other substances of misuse, and may infer a teratogenic effect of methadone.

### Supplementary information

Supplementary Material

## Data Availability

The datasets generated and analysed during the current study are available from the corresponding author on reasonable request.
