# Supplementary material for: Impaired vision in children prenatally exposed to methadone: an observational cohort study
Source: Eye (Lond). 2023 Jul 4;38(1):118–26. doi: 10.1038/s41433-023-02644-3 (PMC10764882; doi:10.1038/s41433-023-02644-3)
Supplement: Supplementary file 1 — Supplementary Material [file 41433_2023_2644_MOESM1_ESM.pdf]

Supplementary Material

Methods for children attending for assessment

Questionnaires

Accompanying adults completed the Strengths and Difficulties Questionnaire [1] and the Behaviour Rating Inventory of Executive Function, 2nd edition [2] to seek evidence of impaired ability to complete visual testing, to be used qualitatively to understand the extent to which any visual problems may have been due to an inability to comply with testing [3].

Visual assessments (detailed results reported elsewhere)

Orthoptic: Monocular and binocular near (Kays letters) and distance (crowded Keeler logMAR) acuity was assessed with and without glasses; additional pinhole testing was performed if acuity was poorer than 0.200 logMAR. Cover tests looked for strabismus (any manifest deviation, i.e. tropia, at near or distance with or without glasses). Binocular single vision (motor fusion) was assessed using prism dioptre base-out tests (20, 15 or 10 prism dioptres). Stereoacuity was assessed with the Frisby Near Stereotest™ (Sheffield, UK). Eye movements (presence of nystagmus, saccades, smooth pursuit, convergence) were assessed clinically. A 'fail' result was pre-determined as acuity poorer than 0·2 logMAR not attributable to refractive error; any manifest strabismus or any nystagmus; inability to overcome any base-out prisms, or a Frisby stereothreshold >110 arcsec. Latent strabismus was noted but not considered a fail criterion. Consenting children who failed orthoptic assessment had pupils pharmacologically dilated for ophthalmic examination.

Eye movement recordings: Children were seated using a chin and forehead rest, with eyes 36 cm from a 53×30 cm (1920×1080 pixel) monitor, wearing any required glasses. Eye images were aligned and focussed to minimise the corneal reflection (EyeLink 1000plus, SR Research Limited, Ottawa, Canada). Following a five point calibration, three recordings of fixation in the primary position were made (both eyes open (BEO), left eye only (LE), right eye only (RE)). Recordings lasted between 10 and 30 s each and were sampled at 500 Hz. Two researchers (DM, EI) masked to all other factors classified the waveforms as in keeping with nystagmus (continuous, rhythmic movement with a slow phase which de-foveated) or normal. Any increase in amplitude and/or intensity with monocular viewing relative to binocular viewing were noted. The direction of the slow phase (left or right) under all viewing conditions and whether the direction switched on occlusion was noted: the presence of this switch in tandem with an increase in amplitude or intensity for monocular relative to binocular viewing was taken to indicate fusion maldevelopment nystagmus syndrome (FMNS). [4]

Facial photography: A medical photographer (AM) took anterior posterior, oblique (3/4) and left lateral views in a clinical studio using a Nikon D610 camera, magnification 1:8/1m on a 105 mm lens, with head alignment standardised in the Frankfurt horizontal plane. A 20 mm sticky scale was placed between their eyebrows. Palpebral fissure lengths were measured using software [5]; two researchers (HM, SB) masked to all other factors ranked philtrum smoothness and lip thinness, reaching consensus by discussion or referral to a third party (RM). Number of sentinel features were noted for each child. [6]

References

1 Goodman R. The Strengths and Difficulties Questionnaire: A Research Note. *J Child Psychol & Psychiat* 1997; **38**: 581–6.
2 Gioia GA, Isquith PK, Guy SC, Kenworthy L. TEST REVIEW Behavior Rating Inventory of Executive Function. *Child Neuropsychology* 2000; **6**: 235–8.
3 Spowart KM, Reilly K, Mactier H, Hamilton R. Executive functioning, behavioural, emotional and cognitive difficulties in school-aged children prenatally exposed to methadone. *Front Paediatr Sec Neonatology* 2023; **11**: doi:10.3389/fped.2023.1118634.

4 Hertle RW, Dell'Osso LF. Chapter 3 Fusion Maldevelopment Nystagmus Syndrome. In: Nystagmus in Infancy and Childhood: Current Concepts in Mechanisms, Diagnoses, and Management. New York: Oxford University Press, 2013: 103–21.
5 Astley S. FAS Facial Photographic Anlaysis Software. 2016. https://depts.washington.edu/fasdpn/htmls/face-software.htm.
6 Scottish Intercollegiate Guidelines Network (SIGN). Children and young people exposed prenatally to alcohol. Edinburgh: SIGN, 2019 Available from URL: http://www.sign.ac.uk.
7 Lipsitz P. Proposed Narcotic Withdrawal Score for Use with Newborn-Infants - Pragmatic Evaluation of Its Efficacy. *Clin Pediatr* 1975; **14**: 592–4.

Figure S1. Flowchart indicating participation of the 153 children from recruitment.

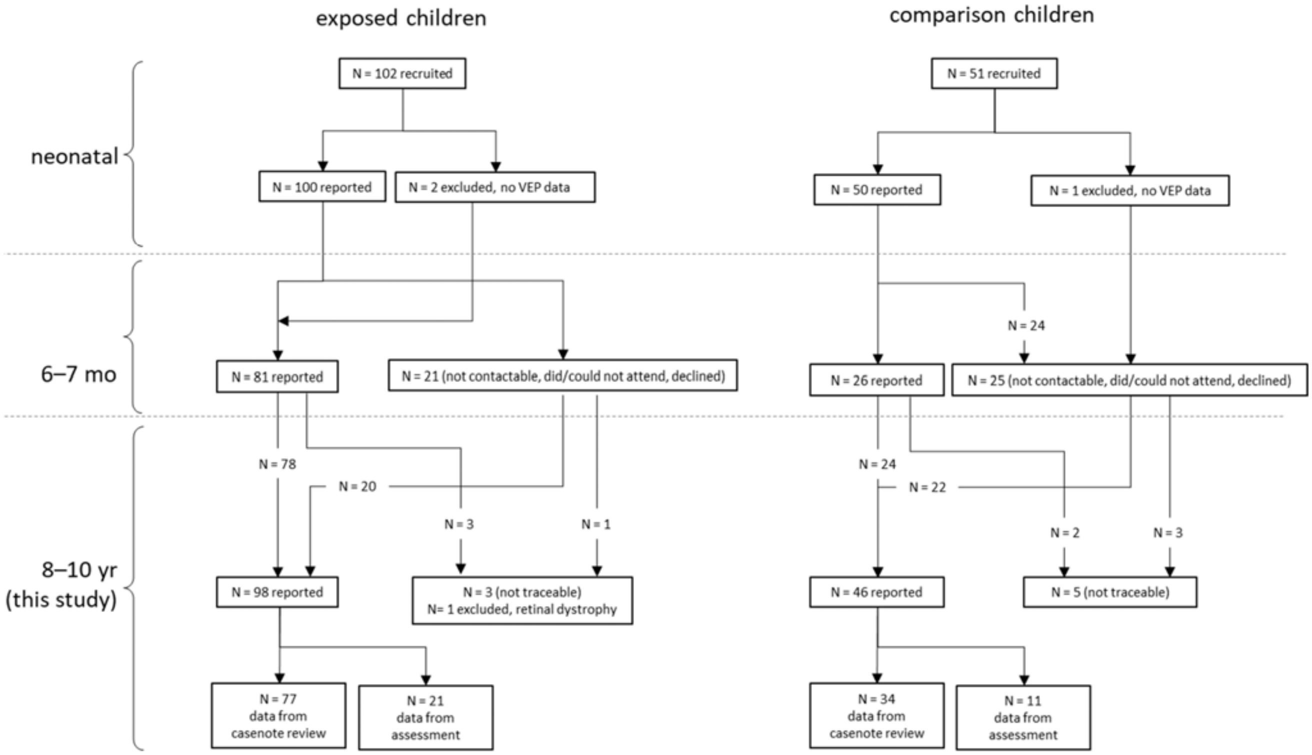

Exposure is defined as methadone exposure during pregnancy; for mothers of 97/98 exposed children methadone was prescribed. Subject #124's mother was prescribed buprenorphine but maternal urine tested positive for methadone and opiates and #124 was included as an exposed child.

Table S1 Comparison of maternal, birth and neonatal characteristics, drug exposure, demographics and outcomes at follow-up for exposed versus comparison children, comparing attending with non-attending children.

	exposed children			comparison children		
	attending (*n*=21)	non-attending (*n*=77)	difference (95% CI), test, *p*-value	attending (*n*=12)	non-attending (*n*=34)	difference (95% CI), test, *p*- value
**Maternal, birth and neonatal characteristics**						
sex, (*n*) % male	(10) 48%	(35) 45%	2 (-20–25) %, χ^2, $p = 0{\cdot}86$	(3) 25%	(18) 53%	-28 (-50–5) % χ^2, $p = 0.09$
gestation, wk [a]	39.4 (37.8–40.4)	39·4 (38·4–40·1)	-1 (-7–4) days, MW, $p = 0{\cdot}61$	39·9 (38·4–41·0)	39·8 (38·3–40·6)	1 (-5–7) days MW, $p = 0.65$
birthweight, g [b]	2878 (448)	2903 (527)	-25 (-258–207), *t*-test, $p = 0{\cdot}83$	3114 (550)	3066 (535)	48 (-337–434) g *t*-test, $p = 0.80$
occipitofrontal head circumference at birth, cm [b]	33.3 (1.8)	33·5 (1·5)	-0.2 (-1.0–0·7), *t*-test, $p = 0.69$	34.1 (1.7)	34.3 (1.5)	-0.2 (-1.4–0.9) *t*-test, $p = 0.68$
maternal tobacco use, (*n*) %	(21) 100%	(72) 94%	6 (-9–14) %, χ^2, $p = 0.23$	(8) 67%	(17) 50%	17 (-15–42) % χ^2, $p = 0.32$
cigarettes per day [a]	10 (10–15)	10 (10–15)	0 (-0–5), MW, $p = 0.57$	10 (10–10)	10 (10–20)	-0 (-10–-0) MW, $p = 0.62$
maternal body mass index [a]	23 (21–25)	24 (21. 5–27)	-1 (-3–0), MW, $p = 0.09$	25 (22–33.75)	23 (21–30.25)	1.5 (-2–6) MW, $p = 0.34$
maternal Carstairs deprivation index [a]	7 (4.5–7)	7 (5–7)	-0 (-0–0), MW, $p = 0.70$	6.5 (5–7)	6 (4–7)	-0 (-0–1) MW, $p = 0.71$
Pharmacologically treated neonatal opioid withdrawal syndrome (*n*) % [c]	(14) 67%	(33) 43%	24 (-0.2–43) %, χ^2, $p = 0.054$	-	-	
**Drug exposure, (n) %**						
methadone	(21) 100%	(77) 100%		0/7 tested	0/21 tested	
dose at delivery (mg/day) [a]	55 (40–80)	50 (30–70)	6.5 (-10–10), MW, $p = 0.36$	-	-	
opiates	(19) 90%	(55) 71%	19 (-2–32) %, χ^2, $p = 0.07$	0/7 tested	0/21 tested	
benzodiazepine	(16) 76%	(50) 65%	11 (-12–29) %, χ^2, $p = 0.33$	1/7 tested	0/21 tested	-14 (-5–51) % FE, $p = 0.25$
cannabis	(13) 62%	(46) 60%	2 (-21–23) %, χ^2, $p = 0.86$	1/7 tested	2/21 tested	5 (-17–42) % FE, $p = 0.59$
amphetamine	(3) 14%	(10) 13%	1.3 (-12–22) %, χ^2, $p = 0.88$	1/7 tested	1/21 tested	10 (-12–47) % FE, $p = 0.44$
cocaine	(4) 19%	(9) 12%	7 (-7–29) %, χ^2, $p = 0.38$	0/7 tested	2/21 tested	-10 (-29–27) % FE, $p = 0.56$
**Demographics and outcomes at follow-up**						
age, yr [a,d]	9.3 (0.7)	7.4 (2.5)	1.9 (1.3–2.6) *t*-test, $p < 0{\cdot}0005$	9.3 (0.7)	7.5 (2.2)	1.8 (0.9–2.6) *t*-test, $p < 0.0005$
birth mother deceased, (*n*) %	(3) 14%	(11) 14%	0 (-13–21) % χ^2, $p = 1$	0	0	
adopted/foster/kinship care	(10) 48%	39/72 known, 54%	-6 (-29–16) % χ^2, $p = 0.60$	(3) 25%	3/29 known 10%	15 (-8–44) % FE, $p = 0.23$
previous hospital eye services	(16) 76%	(50) 65%	11 (-12–29) % χ^2, $p = 0.33$	(3) 25%	(16) 47%	-22 (-45–10) % FE, $p = 0.18$
visual outcome 'fail'	(14) 67%	(42) 55%	12 (-11–32) % χ^2, $p = 0.32$	(2) 17%	(10) 29%	-13 (-33–18) % FE, $p = 0.47$
strabismus	(9) 43%	(37) 48%	5 (-18–27) % χ^2, $p = 0.67$	(1) 8%	(5) 15%	-6 (-23–22) % FE, $p = 0.50$
poor acuity	(7) 33%	(18) 23%	10 (-9–33) % χ^2, $p = 0.36$	(1) 8%	(6) 18%	-9 (-27–19) % FE, $p = 0.40$
poor binocular vision	(8) 38%	(14) 18%	20 (-0.1–42) % χ^2, $p = 0.054$	(2) 17%	(3) 9%	8 (-11–37) % FE, $p = 0.39$
nystagmus	(6) 29%	(14) 18%	10 (-7–33) % χ^2, $p = 0.30$	0	0	
visual detriment index, VDI	4 (0–5)	2 (0–4)	0 (0–2) MW, $p = 0.25$	0 (0–0)	0 (0–2)	

CI, confidence interval; FE, Fisher exact test, MW, Mann Whitney test; OFC, occipitofrontal head circumference. [a]median (inter-quartile range), [b]mean (standard deviation), [c]according to well established hospital protocol [7], [d]age at assessment (*n*=33) or at most recent hospital eye service attendance (*n*=66) or age at most recent healthcare encounter with confirmed local address but no hospital eye service encounters ('pass' vision result attributed, *n*=45).
